# “I dream of an island”: Black joy, storytelling and the art of refusal. Creative methodologies and decolonial praxis in higher education

**DOI:** 10.3389/fsoc.2025.1537033

**Published:** 2025-07-25

**Authors:** Naomi Alormele

**Affiliations:** School of Health Education and Society, University of Northampton, Northampton, United Kingdom

**Keywords:** Black joy, decolonial praxis, Black feminist thought, storytelling, intersectionality in higher education, epistemic injustice, institutional performativity, visual methodologies

## Abstract

This paper advances a decolonial and Black feminist intervention into higher education research by positioning emotive storytelling, creative methodologies, and Black joy as transformative tools for epistemic resistance and institutional critique. Centring the voices of Black women in academic and professional roles across the UK and Canada, the study draws on Decolonial Theory, Black Feminist Thought, and Critical Race Theory to examine how contributors navigate systemic exclusion, racialised emotional labour, and the limitations of performative diversity. Using a cross-contextual, contributor-led approach—including storytelling conversations, reflective journals, poetry, and visual artefacts—this research establishes emotive and creative forms of expression as legitimate and vital modes of knowledge production. Black joy is conceptualised not as an affective state, but as a radical methodological and political framework: enacted through humour, ritual, and care, it becomes a strategy of survival, refusal, and reimagining. Storytelling functions as both method and praxis, offering contributors space to articulate lived realities and assert epistemic agency. Visual artefacts—such as collages, metaphorical drawings, and illustrated poetry—are analysed as counter-narratives that disrupt erasure and reframe Black women’s presence within academic institutions. While UK contributors contend with the afterlives of empire and class-based exclusion, Canadian contributors confront the contradictions of multiculturalism and anti-Indigenous racism. Across both contexts, the study exposes how symbolic inclusion masks structural harm. This study contributes to current debates on decolonising research by demonstrating the power of emotionally grounded, arts-based methodologies to surface hidden forms of knowledge and resistance. It calls for institutions to move beyond rhetorical equity by honouring Black women’s intellectual labour, embedding joy as method, and supporting creative, relational approaches to transformation in higher education.

## Introduction

This paper builds on insights from my doctoral research to examine how emotive storytelling and creative methodologies can advance decolonial praxis in higher education. In my work, I explore decolonial praxis as the application of decolonial theory in transformative actions that dismantle colonial structures of power, knowledge, and representation. This approach prioritises the reclamation of marginalised voices and epistemologies while challenging Eurocentric dominance (Bhambra et al., 2018). By centring the lived experiences of Black women across academic, professional services, and administrative roles, I critique the systemic inequities embedded in institutional structures shaped by colonial legacies. I find that higher education institutions often proclaim commitments to equity and diversity, yet lived realities of Black women reveal persistent barriers rooted in racism, sexism, and exclusion. While much of the existing literature focuses on the experiences of Black faculty and students, my research expands the focus to include the underexplored roles of Black women in wider institutional contexts, where systemic inequities manifest across professional boundaries (Rollock, 2021; Arday, 2022).

Grounded in three intersecting theoretical frameworks, Decolonial Theory, Black Feminist Thought, and Critical Race Theory (CRT), this study uses a multidimensional lens to examine the layered experiences of Black women in higher education across the UK and Canada. Decolonial Theory critiques the enduring influence of colonial power hierarchies in shaping academic structures and epistemologies (Quijano, 2000; Mignolo and Walsh, 2018), highlighting how universities continue to marginalise racially minoritised knowledge systems. This “coloniality of power” manifests in culture, labour, and knowledge production, perpetuating Eurocentric epistemologies (Bhambra et al., 2018; Grosfoguel, 2011). While universities often portray themselves as neutral spaces of knowledge creation, I argue that their curricula, hiring practices, university environments, and leadership structures reflect deep-seated colonial complicity (Almeida, 2015; Smith, 2012).

Black Feminist Thought builds on the decolonial framework by foregrounding the intersection of race, gender, and other identities in shaping the experiences of Black women. Collins (2000) concept of the outsider-within is particularly useful for understanding the liminal and often precarious positions Black women occupy in university spaces.

CRT frames racism as a structural, embedded feature of society rather than an anomaly (Bell, 1992; Delgado and Stefancic, 2017). I align this research with CRT’s emphasis on counter-narratives, which Delgado (1989) and Solórzano and Yosso (2002) identify as essential for exposing the hidden mechanisms of racism and sexism in higher education. Although CRT originated in the U. S. during the 1970s and 1980s in response to the limitations of civil rights law (Bell, 1992), its analytical tools have been extended globally. In the UK, Gillborn (2006) applied CRT to critique systemic racism in education policy, while Dei (1996) drew on CRT to frame anti-racist educational practices in Canada. This diasporic application of CRT, alongside Decolonial Theory and Black Feminist Thought, enables a transnational analysis of how race, gender, colonial histories, and structural violence intersect in academic institutions. It also affirms storytelling as a method of both epistemic resistance and institutional critique—challenging Eurocentric norms while centring Black women’s knowledge and emotional lives (Ahmed, 2017).

I intentionally adopt a decolonial, contributor-led approach that rejects predefined research questions in favour of contributor-driven inquiry. Storytelling emerges in my research as both a methodological and political act of resistance, challenging Eurocentric research paradigms by amplifying the lived experiences of Black women as legitimate sources of knowledge (Smith, 2012; Kinpaisby-Hill, 2020). Reflective journals and photo elicitation complement storytelling by offering contributors flexible and creative means to document their narratives, fostering agency and self-representation throughout the research process. Drawing on McKittrick’s (2021) theorisation of storytelling as a Black intellectual practice, I position these narrative forms as “unorthodox practices of belonging” that honour emotion, creativity, and refusal. By integrating these methods, I align my study with the broader decolonial agenda to dismantle traditional hierarchies of knowledge and foreground marginalised voices in research (Alexander and Mohanty, 1997; Mignolo, 2011).

Through this approach, I offer cross-diasporic insights into how systemic barriers and resistance strategies are experienced by Black women across the UK and Canada (Howard-Hamilton, 2023). In the Canadian context, contributors frequently spoke to the intersections of anti-Black and anti-Indigenous racism, framing their experiences within broader decolonial and settler-colonial struggles. UK-based contributors, by contrast, emphasised how racial marginalisation is compounded by class-based exclusion, underscoring the ongoing impact of British colonial legacies on institutional structures and cultures. My framing draws on a cross-diaspora perspective that acknowledges both the shared experiences and regionally specific dynamics shaping Black women’s lives in higher education. Expanding Gilroy’s (1993) concept of the Black Atlantic, I situate Black women’s identities within institutions marked by the afterlives of slavery and empire, while foregrounding their practices of resilience, refusal, and joy. In doing so I honour the nuanced experiences of Black women working within higher education, rejecting a single story narrative (Adichie, 2009). Although Gilroy’s model has been critiqued for its gender-blindness and limited engagement with intersectionality (Carby, 1999; Wright et al., 2007), I reimagine the Black Atlantic through a Black feminist lens—centering the lived experiences, intellectual labour, and transformative strategies of Black women navigating racial and colonial violence.

I employ emotional labour and Black joy as dual lenses in this study to understand both the burdens and transformative strategies used by Black women in higher education. Emotional labour, a term coined by Hochschild (1983), is particularly pronounced for Black women, who often regulate their emotions to align with institutional norms while suppressing their authentic selves to counter stereotypes such as the “angry Black woman” (Lewis et al., 2017). In contrast, Black joy emerges in my research as a radical act of resistance. Reframing empowerment as a justice-oriented framework, joy challenges institutional indifference and creates spaces of relational accountability and belonging (hooks, 1992; Johnson and MacDonald, 2021; Okello, 2024). Joy, in this context, is not merely a response to adversity but a deliberate and disruptive knowledge practice that confronts structural violence while affirming Black presence and creativity. These intertwined experiences of emotional burden and joyful resistance are most powerfully articulated through storytelling, which I employ not only as a method of inquiry but as a site of political and epistemic intervention.

Ultimately, I position storytelling as a transformative method that critiques systemic inequities while amplifying strategies for resistance, joy, and healing. By bridging individual narratives with structural critique, storytelling illuminates the barriers Black women face in higher education while offering pathways to institutional and epistemic transformation. Through this lens, my research contributes to the broader decolonial agenda of dismantling colonial legacies and creating academic spaces that prioritise justice, care, and the celebration of marginalised identities.

## Methods

### Methodological orientation and research design

This study uses a decolonial, contributor-led qualitative approach grounded in Decolonial Theory, Black Feminist Thought, and Critical Race Theory (CRT). Informed by elements of Participatory Action Research (PAR), contributors were treated as co-creators of knowledge rather than subjects of inquiry, helping shape both the methods and areas of focus (Bless et al., 2013). These frameworks support the centering of Black women’s lived experiences as epistemologically valid, resisting dominant Eurocentric paradigms that often dismiss them as anecdotal or subjective (Collins, 2000; Crenshaw, 1991). Through an adaptive and reflexive research design, I examined how Black women across professional, academic, and administrative roles in higher education navigate exclusion, emotional labour, and resistance in the UK and Ontario, Canada.

This cross-contextual study unfolded in two stages. Stage 1 focused on Black women in the UK and involved three data collection methods: an online survey, storytelling conversations, and reflective journals. Stage 2, conducted in Ontario, Canada, involved adapted storytelling conversations only, reflecting methodological responsiveness to logistical constraints and contributor needs.

In total, 39 contributors engaged in storytelling conversations: 21 in the UK and 18 in Ontario, Canada. The UK sessions comprised of six online group conversations involving 2–4 contributors each, while the Canadian phase included nine sessions delivered both in person and online, including four one-to-one conversations. Six UK contributors also submitted reflective journals, offering extended multimodal engagement. Prior to these sessions, an anonymous online survey was conducted in the UK. Open for four weeks, it gathered 101 responses from staff across 55 universities. The survey included both quantitative and qualitative items, though analysis focused on the qualitative data to shape the emotional vocabulary of subsequent methods.

The UK and Ontario were chosen for their distinct colonial legacies and shared diasporic entanglements. This research was originally funded as a UK-based project; however, upon receiving additional funding to support international fieldwork, I selected Canada as a second research site. Ontario, bordering the U.S. and historically connected to the Underground Railroad, has deep transatlantic ties and a strong presence of Black and Indigenous scholars (Henry et al., 2017). Literature gaps on Black women in Canadian academia, as well as practical concerns about political coherence across U.S. states, shaped the decision to focus on Canada instead of expanding to the U.S.

### Survey: foundational mapping and contributor vocabulary

The UK-based survey provided foundational insights into contributors’ identities and experiences within higher education, shaping the thematic focus of subsequent qualitative methods. Guided by a decolonial focus, the survey emphasised open-ended responses, allowing contributors to articulate their perspectives without being confined to predefined categories (Smith, 2012). While the survey was not conducted in Canada, its findings informed the structure and emotional vocabulary of the storytelling prompts used in both contexts. This approach ensured alignment with the study’s decolonial ethos, prioritising contributors lived experiences (Mignolo and Walsh, 2018; Kovach, 2009).

### Storytelling conversations: emotional resonance and communal insight

Storytelling was central to this project as both a method and a mode of resistance. Grounded in CRT’s emphasis on counter-narratives and Black Feminist Thought’s validation of lived experience, these sessions offered contributors space to reflect on safety, marginalisation, and joy (Collins, 2000; Delgado, 1989; Spencer, 2016). Adhering to Principled Space guidelines (BARC Workshop, 2024), I emphasised confidentiality, belief in contributors’ experiences, and equitable participation (Tronto, 1993; Ahmed, 2012). In all conversations, contributors were invited to respond to emotion-based prompts such as “safe,” “vulnerable,” “judged,” or “empowered.” In Canada, conversations were adapted to include more direct invitations to discuss Black joy after early sessions revealed the emotional toll of unprompted storytelling. This methodological evolution, prompted by emotional intensity and guided by a Black feminist ethic of care (Tronto, 1993), enabled contributors to define joy on their own terms, expanding the study’s focus beyond trauma. The joy-focused prompts deepened the range of data and aligned with the decolonial imperative to centre not only pain but also pleasure, resilience, and radical hope (Love, 2019; Campt, 2017).

### Reflective journals and creative expression

Only used in the UK phase, reflective journals offered contributors time and space for introspective expression beyond the temporal bounds of conversation. Contributors were again not provided with questions to answer for this journal, but instead guidance around things to consider such as safety/vulnerability. This research output allowed for written, visual, and poetic engagement with emergent themes such as masking, hypervisibility, and belonging. Some contributors used images to express aspects of their experience, creating visual metaphors for their professional realities. For example, the metaphor of a mask (Celeste, UK-RJ) captured the tension between visibility and silencing. Poetry was also used as a form of creative self-expression, capturing themes like hypervisibility and belonging in ways that traditional prose could not. These journals deepened understanding of emotional labour and internalised exclusion, while honouring contributors’ creative autonomy (Leavy, 2018; McKittrick, 2021). Journaling became both a form of intellectual resistance and emotional healing, demonstrating the value of creative methods in challenging colonial ways of producing knowledge. By using these methods, the study respected the contributors’ choices and provided a space where their unique ways of expression were valued. This approach supported the study’s focus on decolonial and feminist principles, ensuring that contributors’ voices were not just acknowledged but fully appreciated in all their richness.

### Inclusion of visual artefacts as methodological practice

In keeping with the study’s decolonial and Black feminist methodological grounding, visual artefacts produced by contributors, such as artwork, collages, and poetry, are included directly within the body of this article. Their integration is not merely illustrative but a core component of the methodological approach, recognising creative expression as a valid and rich form of data. This aligns with calls from decolonial and feminist scholars to challenge Eurocentric norms of research representation (Collins, 2000; Smith, 2012; Leavy, 2018). Visual artefacts are positioned as acts of storytelling and resistance, offering insights into contributors’ emotional, intellectual, and professional lives that might otherwise remain inaccessible through text alone. Including these artefacts within the main body of the article honours contributors’ agency and affirms multimodal storytelling as central to the study’s epistemological commitments.

### Adapting methods across contexts

The comparative framing across the UK and Canada is central to this study. While themes such as emotional labour, hypervisibility, and performative allyship appeared across both sites, regional distinctions emerged. In Canada, contributors explicitly named anti-Indigenous racism and the contradictions of multiculturalism, while UK contributors connected exclusion to austerity, classism, and empire. These differences shaped how contributors narrated their experiences and how I adapted methods.

For instance, in the Canadian phase, the absence of journals and surveys was mitigated by building on emotional vocabularies developed in the UK and by introducing explicit joy prompts to redress emotional exhaustion. This reflexivity ensured that contributors remained central in shaping the research process, and that the project honoured their emotional labour while expanding methodological richness.

### Data analysis and ethical practice

Each storytelling session and journal was first analysed individually to centre contributors’ voices before identifying overarching patterns. These themes included symbolic inclusion, masking, institutional betrayal, and Black joy as fugitive practice. These were then aligned with the principles of intersectionality and double consciousness, identifying how race, gender, class, neurodivergence, and other factors intersected in contributors’ experiences.

Contributors self-selected pseudonym names to preserve confidentiality while ensuring transparency. Contributors chose their preferred methods and modes of interaction. Emotional wellbeing was prioritised through the inclusion of breaks, check-ins, and access to support resources when needed.

As a Black British Ghanaian researcher in higher education, I approached this work with deep reflexivity. My positionality was a source of both insight and accountability (Adu-Ampong and Adams, 2020). Trust was often grounded in shared experience, yet I remained aware of power dynamics in research relationships. I positioned myself as a co-collaborator rather than an extractor of knowledge, aligning with decolonial critiques of conventional research hierarchies (Mignolo and Walsh, 2018; Smith, 2012). In line with feminist ethics of care (Tronto, 1993) and supported by Bergland (2021) critique of neoliberal research cultures, I resisted extractive modes of inquiry by embedding care, flexibility, and attentiveness throughout the research process.

Importantly, I extended this ethic of care to myself. Engaging with stories of racial trauma, exclusion, and institutional betrayal demanded emotional labour and vulnerability. In response, I incorporated self-care practices such as reflective journaling, debriefing with trusted peers, and setting emotional boundaries to minimise burnout. Caring for my contributors meant also caring for the conditions that enabled me to hold space for their stories with empathy, presence, and integrity.

### Findings and analysis

#### Navigating invisibility and double consciousness

Contributors’ narratives repeatedly reflected a dual burden: the emotional labour of being hypervisible as symbolic representatives of diversity, and simultaneously invisible in decisions, recognition, and institutional support (Ahmed, 2012). This paradox, being both seen and unseen, illustrates what Du Bois (1903) terms double consciousness, and what Collins (2000) conceptualises as the outsider within. Together, these frameworks illuminate the emotional and epistemic labour that Black women endure to survive institutional spaces shaped by whiteness and patriarchy.

This is demonstrated in Linda’s (UK-RJ) reflective journal, anchored by the metaphor of being a “superwoman with a smile,” which articulates the painful negotiation between her professional self and internal identity. She writes:

“In an effort to not make others fear my Blackness… I put on a smile—to demonstrate that I am approachable, to allow them to feel that with me they can be safe.”

This statement reflects the internalisation of double consciousness—Linda must constantly monitor and regulate herself to appear non-threatening, palatable, and emotionally manageable for her white colleagues. Her metaphor of wearing a cape evokes heroism but also exhaustion:

“The days where I can no longer wear a cape or mask… I hide away in my superhero refuge which is my home and family.”

Here, home is the only space where her full self can safely emerge. Linda’s experience powerfully illustrates Collins (2000) outsider within, and the racialised pressure to perform strength without vulnerability. This protective, constant masking takes a toll—what Kinouani (2021) describes as the psychological and physiological consequences of racial trauma, where the sustained stress of navigating white institutional spaces profoundly impacts Black individuals’ wellbeing.

Similarly, Celeste’s (UK-RJ) journal draws on visual metaphor to describe her experience using the image of a mask split in two. The left-facing side, she writes, represents her efforts to assert her Blackness and intersectional identity, despite being “part of a blended discourse” where her message becomes “safely contained.” The right-facing side symbolises her erasure beneath “hegemonic domes of institutional Whiteness.” She writes:

“In that deeper, darker life-world… my identity becomes muted within an oppressive framework… There is no place for me to be heard there.”

Celeste’s duality reveals the institutional demand for conformity that renders authenticity risky. Her description evokes the psychological strain of being either “coached out of the shadows” or “mentored while in the light,” a conditional visibility that serves white comfort and control.

Together, these accounts challenge the celebratory narratives of “diversity” by exposing how inclusion is often contingent upon the emotional labour and behavioural modulation of Black women. Ahmed (2012) argues that institutional diversity frameworks reward the “safe” Black woman, one who does not appear “too radical,” too resistant, or too honest about exclusion. Linda’s and Celeste’s reflections make visible how strategic self-censorship is both expected and rewarded, creating a hollow form of inclusion that ultimately reinforces marginalisation.

These findings show that double consciousness is not simply a psychological state, it is structurally produced and institutionally sustained. Black women in the neo-liberal academy must continually negotiate between visibility without agency and invisibility as protection. Their resistance, whether through metaphor, silence, or artistic expression, demonstrates the ongoing cost of institutional belonging and the urgent need to transform what inclusion really means (Williams, 2023).

Contributors such as Rosie (CA-C), Mariah (UK-C), Destiny (CA-C), and Kara (UK-C) described environments where they were expected to conform to tightly regulated emotional expectations. Mariah’s (UK-C) strategic use of a “hyper-bubbly” persona to avoid being labelled “angry” exemplifies the racialised performance of emotional labour. Kara’s (UK-C) narrative of being scrutinised for her appearance, and Destiny’s (CA-C) reference to the frequent hyper sexualisation of Black women, expose the ways in which gendered and racialised stereotypes impose constraints on how Black women are perceived and treated (Daniel, 2018).

### Emotional labour, masking, and institutional non-performativity

Across the UK and Canadian data, emotional labour emerged as a central mechanism through which Black women survived institutional spaces that simultaneously demanded their presence and denied their power. For Black women in higher education, this labour is racialised, gendered, and deeply tied to histories of care, silence, and survival. Linda (UK-RJ) characterised this as wearing “invisible armour” to shield herself from constant scrutiny and racial microaggressions. “Every day, I put on my cape to shield myself from the whispers, the stares, the expectations to prove my worth,” she explained. Her drawing of a Black woman wearing a superhero cape nodded to the “strong Black woman” trope, which frames resilience as both a strength and a burden (Beauboeuf-Lafontant, 2009).

Amber (UK-C) echoed this theme, describing the cumulative toll of racial violence: “It’s exhausting to always have to correct and educate.” Meanwhile, Rosie (CA-C) likened her experience to being confined in a “box,” restricted by institutional structures that suppress Black voices. “You’re restricted… people do not like to hear from Black people too often,” she explained, reflecting the coloniality of power (Quijano, 2000) that limits Black professionals to predefined roles while silencing their full participation (Tuck and Yang, 2012).

Contributors also described being positioned as emotional anchors, expected to support students, mediate tensions, lead EDI initiatives, or offer informal pastoral care, often without recognition or reward. As Linda’s (UK-RJ) account illustrates, emotional labour also involved managing others’ discomfort with Blackness:

“In an effort to not make others fear my Blackness… I put on a smile—to demonstrate that I am approachable, to allow them to feel that with me they can be safe.”

Linda’s self-regulation exemplifies the suppression of authentic emotion to avoid being framed as “aggressive” or “angry,” a phenomenon rooted in pervasive stereotypes. The expectation to remain emotionally composed, even in the face of microaggressions, appropriation, or institutional betrayal, creates a psychological burden that is both isolating and exhausting (Collins, 2000).

This burden is further intensified by the need to code-switch and mask, behaviours that contributors linked to survival. Code-switching refers to adjusting one’s language, tone, or expression to align with dominant (white, middle-class, neurotypical) norms (Bell et al., 2020). For neurodivergent Black women in particular, this often entailed an additional layer of labour, concealing traits that deviated from behavioural norms, suppressing cultural identity, and enduring constant self-monitoring (Lewis and Arday, 2023). These behaviours are not simply social adjustments, they are protective strategies shaped by the need to avoid punishment or exclusion.

Nathalie (UK-C) captured this tension vividly:

“I’m raging inside, but I cannot show it. Because if I do, they’ll say I’m being unprofessional, or I’m not a team player.”

Her reflection illustrates what Du Bois (1903) described as double consciousness, and what Kinouani (2021) and Smith (2012) reference as racial battle fatigue and the psychological wear and tear of enduring racialised environments. This fatigue was not only emotional but epistemic: contributors described the exhaustion of explaining, justifying, or defending their experiences to institutions that demanded evidence of harm but failed to act on it.

Vanessa (CA-C) similarly reflected on the emotional regulation required to maintain a sense of “professional composure” amid ongoing marginalisation. “It’s exhausting to keep smiling, to keep editing myself… just to be heard without being punished,” she explained. Her comments illuminate the paradoxical demands placed on Black women to perform gratitude and patience in institutions that continuously ignore their expertise.

Ahmed’s (2012) concept of non-performative diversity work is particularly salient here. She argues that institutions often *perform inclusion* without enacting structural change. Contributors’ accounts reflected this dynamic. Ruth (CA-C) reflected:

“I do not know about the word safe… but I have cultivated a brave space.”

Her distinction between “safe” and “brave” aligns with Arao and Clemens (2013), who argue that “safe space” is often a euphemism that protects institutional norms rather than those most harmed by them. Ruth’s insight underscores that emotional safety is not granted by institutions, it is forged by those navigating them.

This performativity was also evident in accounts of epistemic violence. Adrienne (CA-C) described how her ideas were only taken seriously when repeated by white colleagues. This reflects Fricker’s (2007) notion of testimonial injustice, in which credibility is unequally distributed, and Dotson’s (2011) concept of epistemic oppression, where marginalised people are excluded from knowledge production.

These dynamics are compounded by colonial legacies. In Canada, the myth of multiculturalism obscures systemic racism and casts Black women as perpetual outsiders (Maynard, 2017; Medovarski, 2019). In both contexts, contributors experienced emotional labour as a form of containment, performing strength and care while their contributions were erased or co-opted. The labour extended beyond institutional roles to self-preservation: Celeste’s (UK-RJ) metaphor of the mask symbolised the cost of being authentic in a space where identity had to be managed to ensure survival.

“In that deeper, darker life-world… I have no visage, shape or form in such spaces.”

Celeste’s words reflect how safety is not simply about the absence of threat but the freedom to be visible, to speak, and to be heard. This aligns with the evolving terminology explored across research outputs, where contributors frequently rejected institutional definitions of safety and redefined it through the lens of resistance, refusal, and relational care.

### Systemic exclusion and the daily realities of racial violence

While emotional labour often operates at the interpersonal and symbolic levels, contributors also described concrete experiences of systemic exclusion and institutional racism that shaped their professional environments.

The contributors’ narratives also revealed deep-rooted inequities in academic spaces, where systemic racism and exclusionary structures persist despite institutional commitments to equity and diversity (Mirza, 2018). Amber (UK-C) described the initial empowerment she felt in her professional services role, which soon turned into frustration as she encountered escalating incidents of racial violence. Experiences such as being mistaken for another person of colour or subjected to invasive comments, such as “Can I touch your hair?” eroded her sense of belonging and professional identity. She recounted being “purposely embarrassed or humiliated in front of colleagues,” which left her increasingly isolated and emotionally drained. “It’s like carrying an extra weight that my colleagues do not even realise exists,” she explained, underscoring the psychological toll of navigating exclusionary environments.

Vanessa (CA-C), who worked in student services, described the exhaustion of being positioned as both a symbolic figure and an emotional resource. “I became the face of diversity,” she reflected, “but not the voice.” Her narrative revealed a recurring tension between visibility and agency, where her professional expertise was undermined by colleagues who saw her primarily as a representative of race, not as a peer. Vanessa also detailed how even her attempts to suggest policy improvements were dismissed or reframed as emotional outbursts: “Suddenly I’m the angry Black woman… not someone bringing forward valid, strategic concerns.” This form of institutional gaslighting, where contributions are systematically devalued or misread through racialised stereotypes (Opini and Henry, 2022). These seemingly minor acts are not isolated but represent systemic patterns of exclusion that perpetuate institutional racism (Lewis et al., 2017). Similarly, Celeste (UK-RJ) described how hierarchical mentoring structures reinforced white hegemony, compelling her to conform to institutional norms. These dynamics exemplify the performative allyship embedded within academia, where tokenistic diversity gestures fail to address structural inequities.

In contrast, supportive mentoring relationships with other Black women within and outside of the institution emerged as a counterpoint to these experiences. Celeste (UK-RJ) described these relationships as “reciprocal, sensitive, and open,” fostering a sense of “struggle-in-common” that created psychological safety. This contrast between exclusionary and inclusive spaces highlights the potential for transformative relationships that centre solidarity and mutual empowerment.

### Comparative reflections and diasporic resistance

While institutional dynamics varied between the UK and Canada, the emotional labour, exclusion, and strategic resistance described by contributors reveal a shared geography of oppression and reimagination. Du Bois (1903) concept of *double consciousness* was powerfully echoed in both regions, as contributors balanced their own self-definition with the institutional roles imposed on them. Whether negotiating racialised expectations of femininity, leadership, or gratitude, they experienced a persistent duality: visibility without power, inclusion without safety (Chance, 2022).

Yet the data also showed important contextual differences. In Canada, contributors frequently referenced the ongoing legacies of settler colonialism and anti-Indigenous racism, drawing connections between their own marginalisation and the broader racialised landscape of Canadian higher education. Scholars like Smith (2012) and Quijano (2000) offer a decolonial framing that helps explain how this awareness emerges not just from individual experience, but from a broader engagement with Indigenous resistance and the academic disciplines of Black and Indigenous Studies. In contrast, UK contributors referenced colonial legacies more implicitly—often framed through class, austerity, or empire—but felt their experiences were equally shaped by Britain’s imperial past (Akala, 2018). Contributors like Amber (UK–C) and Leah (UK–C) described informal networks as critical buffers against racial gaslighting and symbolic roles.

This comparative lens helps illuminate how colonialism’s afterlives continue to shape both institutional culture and the narratives contributors use to survive within it. Institutional responses to racism were viewed by contributors in both countries as largely performative, echoing Ahmed (2012) critique of diversity as non-performative speech. The symbolic nature of equity statements and initiatives, particularly after global events like the murder of George Floyd, left contributors hyper visible yet unsupported, carrying the burden of institutional change without the power or resources to enact it.

Mentorship emerged as a critical site of both emotional labour and transformation. Where these institutions often fell short, contributors leaned into informal networks, personal boundaries, and quiet refusals as modes of resistance. In Canada, where institutional race networks were described as sparse or ineffectual, contributors like Rebecca (CA–C) described a personal ethic of intergenerational accountability: *“I know whenever I leave this space… someone remembered what I may have gone through.”* These echoed similar commitments in the UK, where sisterhood often emerged through precarious or self-initiated networks.

Additional Canadian contributors echoed the emotional toll of navigating whiteness while building care-based counterspace, consisting of informal peer groups that offered validation, shared cultural knowledge, and space for vulnerability. Deandra (CA–C), for example, spoke of the exhaustion of “always having to defend or translate” her experiences, noting how informal peer groups provided emotional grounding that was otherwise missing in formal mentorship structures. Similarly, Noelle (CA–C) reflected on the importance of “non-white spaces where I do not have to explain myself”—spaces of rest, solidarity, and resistance. These accounts reflect what Tichavakunda (2021) describes as the formation of racialised counter spaces, where the labour of belonging is eased and emotional survival becomes possible.

### Intersectionality and identity: amplifying layers of exclusion

The contributors’ narratives revealed how intersects of race, gender, and class amplified their marginalisation within academic institutions. Celeste (UK-RJ) described her experience as a mature, working-class Black woman as a constant negotiation of her “dual existence,” where she reconciles her authentic identity with external perceptions imposed upon her. “I am constantly seen as an outlier—never neutral, always a deviation,” she wrote, aligning with Crenshaw (1991) framework on how overlapping identities intensify marginalisation.

Linda (UK-RJ) offered a contrasting perspective, framing her multifaceted identity as a source of empowerment: “My race, my age, my gender—they are all seen as disadvantages, but together, they are my power,” she stated. This sentiment highlights the contributors’ resilience in reclaiming agency despite systemic barriers.

Meanwhile, Yasmin (UK-R) reflected on the loss of a supportive and inclusive work environment. She described how, in her early career, being part of a predominantly Black team fostered a sense of safety and collaboration: “If I made a mistake, they would help me to sort it out.” However, as key team members left, she found herself increasingly exposed to the biases embedded within the institution, underscoring the fragility of inclusive spaces within exclusionary systems.

### Performative allyship, complaint, and institutional violence

Contributors in both the UK and Canada consistently described experiences of betrayal and harm resulting from institutions’ superficial commitments to racial equity. Despite stated commitments to diversity, the everyday realities of many Black women reflected what Ahmed (2012) terms non-performative institutional speech acts, statements about inclusion that do not lead to meaningful action. These moments were not isolated missteps but indicative of deeper patterns of institutional violence that invalidate, erase, or co-opt Black women’s contributions. Contributors described a dissonance between public-facing commitments to anti-racism and the internal resistance they encountered when raising concerns or proposing structural changes. This is particularly evident in Adrienne’s (CA-C) account of epistemic appropriation, where her ideas were only credited when voiced by white colleagues:

“It wasn’t until someone else repeated my idea—someone white—that it became something the department could get behind.”

This dynamic reflects what Fricker (2007) defines as testimonial injustice and links to Dotson’s (2011) epistemic oppression, in which marginalised individuals are systematically disqualified from being recognised as knowers. For Black women, such appropriation not only invalidates their intellectual labour but also reinforces institutional hierarchies in which whiteness remains the default voice of authority. This is also reflected in Amber’s (UK-C), account reflecting on her role in professional services, she expressed frustration at being tokenised for her visibility while excluded from decision-making processes. “I feel like a box-tick exercise,” she noted, encapsulating the dissonance between institutional rhetoric and lived realities.

Lesly (UK-RJ) described the guardedness she adopted as a form of self-preservation against colleagues who professed allyship but failed to provide meaningful support. This guardedness, she explained, was necessary to protect her mental health and navigate the “social and political warfare” of academia, echoing Lorde’s (1984) call for radical self-care and strategic refusal in the face of institutional betrayal.

This performative inclusion also extended to symbolic initiatives such as charters, awards, and EDI statements. While many contributors had been involved in such efforts, they were often disillusioned by the lack of follow-through. The emotional cost of participating in these processes, only to see minimal or superficial outcomes, left many feeling disposable. Jane (CA-C) highlighted the dissonance between institutional commitments to anti-racism and the realities faced by Black staff. Reflecting on her department’s post-Black Lives Matter policies, she observed, “These frameworks are often designed to manage optics rather than implement change.” In this Jane makes reference to how the language of inclusion can often become a tool for concealing exclusionary practices (DiSaia et al., 2023). This performativity reinforces systemic racism by prioritising institutional image over substantive equity, placing the burden of advocacy on Black women while denying them meaningful agency in shaping institutional reforms (Kendi, 2019).

These patterns of invalidation often escalated when contributors attempted to raise formal complaints. Multiple contributors described the weaponisation of complaints procedures, where reporting racism or marginalisation led to further harm. Instead of being taken seriously, contributors were often silenced or framed as difficult. This aligns with Ahmed (2012) critique of the complaint process as a “brick wall,” where the burden of proof lies with the complainant, and the process itself becomes a source of trauma.

In UK conversations, contributors reflected on the limits of institutional accountability. Erin (UK-C) observed that even when individuals witnessed or experienced racism, “the power hierarchies are so evident… it’s kind of impossible to penetrate that and their complaint goes nowhere.” Her insight underscores how institutional structures protect seniority and status over justice. In the Canadian context, Lesly (CA-C) described the silencing effect of complaint processes: “If I talk or… complain, it looks like I’m ruffling feathers… like, well, you cannot do that, right? You might get kicked out. And so I’m being silenced, right, because of my identity and because of my positionality in academia.”

Cindi (CA-C) described taking a leave of absence following a “well-orchestrated harassment complaint” brought against her by faculty members and amplified by a white supremacist organisation. “Silenced,” she said simply, before asserting: “What I am is not afraid… I took a leave of absence as a mechanism of empowerment… If my presence is deemed valuable to the institution, let them feel my absence.” Her account exemplifies how refusal and strategic withdrawal can function as counter-institutional resistance, reclaiming agency in contexts where formal mechanisms fail to offer protection or redress.

These accounts expose how complaint becomes a site of institutional violence, where the emotional labour of surviving racism is compounded by the administrative burden of documenting it. Moreover, the failure of institutions to respond meaningfully to complaints reflects interest convergence (Bell, 1992)—the idea that progress for racially minoritised groups is only permitted when it aligns with the interests of those in power.

### Black joy as resistance, reclamation, and method

Black joy emerged not only as a theme in contributors’ narratives but as a methodological and decolonial strategy, a fugitive practice of resilience, reclamation, and refusal. Building on bell hooks (2000) framing of joy as love and self-love, contributors described joy not as a distraction from struggle, but as central to their survival and resistance within institutions that routinely denied them emotional safety. Expressed through poetry, laughter, cultural rituals, storytelling, and care, joy became a collective strategy to resist dehumanisation and reclaim presence.

Deandra (CA-C), reflecting on her journey within academia, shared how connecting with other Black women in her department helped her “relearn joy” after years of isolation and microaggressions. “We would laugh in meetings just because we could. It felt like breathing again,” she explained. These small yet radical acts of communion and care became essential to her survival. D (CA-C), reflecting on her experience in Canada, emphasised how moments of shared joy and community offered critical emotional and professional support, enabling her to navigate institutional barriers with greater resilience. Similarly, in her reflective journal, Celeste (UK-RJ) wrote, “We understand the struggle-in-common,” highlighting how relationships with other Black women cultivated a shared sense of belonging and strength. These insights foreground the importance of sisterhood and solidarity, what contributors consistently referred to as lifelines of support, mentorship, and empowerment in environments that sought to isolate or undermine them.

These narratives affirm that joy is deeply relational. Crucially, this joy was not confined to individual feeling—it was relational, communal, and strategic. Black joy surfaced in what Tichavakunda (2021) refers to as “positive racialised emotions,” the shared pride, recognition, and celebration that counter racialised marginalisation in historically white institutions. While his focus is on student experiences in the US, contributors to this study described similar dynamics within staff and academic communities across the UK and Canada.

From a methodological perspective, Black joy also reshaped the research process itself. After early Canadian interviews, I adapted the format to introduce joy as an explicit prompt, an evolution grounded in a feminist ethic of care and contributor feedback. Rather than directing the conversation, I created space for contributors to reflect on what joy meant to them, verbally naming Black joy while allowing responses to emerge organically. This process resonated with Black feminist epistemologies that foreground the full emotional landscape of lived experience, not just its pain (hooks, 1992; Love, 2019).

This move away from purely deficit-based narratives aligns with the call to centre joy, care, and agency in critical qualitative research. In line with my thesis, this paper positions Black joy not as a retreat from systemic critique but as an epistemic intervention—a knowledge practice that insists on Black presence, creativity, and brilliance (Okello, 2024). Joy, in this sense, is not just an outcome of resistance, it is resistance.

One of the most poignant examples of joy-as-resistance came through Beauty in Flight’s (UK-RJ) reflective journal submission, a poem titled “The Many Hats I Wear.” This piece encapsulates the dual burden of hypervisibility and invisibility faced by Black women in academic institutions, while simultaneously asserting agency and joy as radical acts:

The Many Hats I Wear

Beautiful, Intelligent, Resilient, Black Woman

Continuously climbing and looking over the wall

Not going to sit and cry anymore

I will keep pushing and climbing to the top

At some point, finally yes you will see me

If not, you will hear me or hear of me

My voice and presence cannot be ignored or silenced anymore

Beautiful, Intelligent, Resilient, Black Woman

Yes, I am capable of achieving higher knowledge and academia

My experience and expertise matter

And yes, sometimes I am passionate, not angry

Yes, at times I am emotional, not a drama queen

Yes, I am tired of constantly trying harder, pushing harder, and being asked to do better

The poem is both a powerful assertion of identity and a reclamation of agency, positioning lived experience, cultural pride, and emotional truth as valid forms of intellectual expression. Its refrain, “Beautiful, Intelligent, Resilient, Black Woman,” serves as an affirmation and a counter-narrative, challenging stereotypes that have historically framed Black women as inferior, overly emotional, or invisible. The transition from frustration (“Yes, I am tired”) to defiance (“I will keep pushing and climbing to the top”) mirrors Lorde (1984) framing of joy as a radical act of resistance, transforming a narrative of marginalisation into one of empowerment.

The poem exemplifies what hooks (2000) describes as a “loving self-regard” and what Lorde (1984) frames as joy rooted in resistance. In refusing deficit framings and reclaiming the narrative of emotional expression, Beauty in Flight confronts the racialised and gendered tropes, such as the “angry Black woman” or “drama queen,” that so often constrain Black women’s authenticity. The poem becomes, in this context, a creative fugitive act (Moten, 2003; Campt, 2017), one that defies erasure through rhythm, voice, and pride.

What strikes me particularly about the poem is its articulation of the “many hats” contributors wear, teacher, mentor, poet, mother, while navigating heightened scrutiny and resisting erasure. Beauty in Flight’s verse not only calls for visibility in her statement; “At some point, finally yes you will see me,” but also rejects tokenistic diversity by demanding recognition on her own terms. Her creative expression illustrates how contributors use storytelling as an intellectual and emotional strategy to resist systemic inequities, reclaim joy, and assert their presence in spaces that often marginalise them.

This expression aligns with the wider findings of this study, where contributors used poetry, storytelling, and visual art not only to narrate pain but to craft visions of joy, belonging, and reclamation. Creative methodologies allowed contributors to be heard on their own terms, transforming lived experience into forms of knowledge, resistance, and care. Grace (UK-RJ) described her artwork as “my way of saying, ‘I’m here, and I belong, on my own terms,’” while Celeste’s (UK-RJ) used imagery and symbolism to depict her journey through exclusion and empowerment. These creative outputs challenged traditional academic norms, privileging lived experiences and emotive knowledge as valid forms of resistance.

For Black women working in academic spaces, joy is not merely a personal experience but a deliberate and collective act of defiance against institutional structures that seek to marginalise them. This aligns with Ruth’s (CA-C) reflection, where she speaks of longing for an “island”—a space where joy and serenity are not just fleeting moments but integral to the experience of being and thriving in university spaces.

Black joy was also spatially and contextually situated. In the UK, joy, referenced as self-care, was often framed as “hard-won,” emerging in spite of class-based elitism, surveillance, and austerity politics. In Canada, contributors reflected on the affective boundaries imposed by “polite” racism and multicultural erasure but described finding joy in cultural hybridity and intergenerational knowledge.

Significantly, the pursuit of Black joy, while present in both contexts, surfaced differently. In the UK, contributors often offered unprompted reflections on joy as cultural pride, shared humour, and small moments of power. In Canada, intentional invitations to reflect on joy were often necessary to surface these narratives. This difference points not to a lack of joy, but to the importance of creating emotional safety and narrative space for joy to be acknowledged, remembered, and named.

Across both contexts, Black joy was expressed not as naïve optimism but as a method, a form of fugitive resistance, and a vision of what might yet be possible. It was embedded in mentorship, artistic expression, community rituals, and quiet refusals to be silenced. These findings reinforce Black feminist assertions that joy is not secondary to struggle—it is a central strategy of survival, a radical mode of care, and a refusal to be reduced to pain (hooks, 1992; Campt, 2017; Okello, 2024).

### Mentorship, sisterhood, and epistemic safety

Alongside these practices of joy and resistance, contributors highlighted the importance of relational strategies, particularly mentoring and sisterhood, as tools for survival and empowerment within exclusionary institutions (Osho and Alormele, 2024).

Mentorship emerged as a recurring theme in the narratives, offering both challenges and opportunities for Black women in academic spaces. Celeste (UK-RJ) described her interactions with White mentors as a “performative dance,” where rigid hierarchies often reinforced exclusion rather than fostering growth. In contrast, her relationships with Black women mentors were described as empowering, characterised by mutual respect and understanding. “We graciously mentor each other into a place of psychological safety,” she reflected, emphasising the role of sisterhood in creating spaces of belonging.

D (CA-C) highlighted the transformative impact of being assigned a Black faculty mentor upon joining her department. This mentorship provided her with “the language to advocate” for herself and manage her workload effectively, preventing burnout. Natalie (UK-C) underscored the importance of seeking external networks when internal support was lacking. “These networks provide me with the tools and connections I need to thrive,” she explained, highlighting the proactive strategies Black women adopt to counteract systemic barriers.

Empress (CA-C), reflecting on her early career as an EDI hire, described a guarded optimism rooted in cautious trust: “I always come into the work knowing that the Academy wasn’t built for someone like me in the first place… I’m still adamant about creating a different model for myself that’s more sustainable.” Her insights reflect a deliberate strategy of building community and seeking joy without overextension.

Noelle (CA-C), now a senior faculty member, echoed this concern, emphasising the emotional toll of institutional betrayal: “When difference comes in the door, there’s a lack of resources. There’s a vision about Black scholars, but not for them.” She described the joy and affirmation she found in mentoring a PhD student writing about Black joy: “It was amazing… a reminder of why I do this work.” These accounts highlight not only the emotional sustenance provided by mentorship, but also its role in creating epistemic safety, spaces where Black women can be fully seen and valued across generations of scholarship.

These accounts illustrate how mentorship and sisterhood play a vital role in countering institutional exclusion. Through these relationships, contributors found psychological safety, professional growth, and a sense of community, challenging the isolation that systemic inequities often perpetuate.

### Creative artefacts—visualising black joy and resistance

The reflective journals submitted by contributors provided not only written reflections but also a powerful collection of visual artefacts. These artworks, selected and described by contributors, speak directly to the emotional, psychological, and institutional experiences of Black women in academia. Including a selection of these artefacts in the body of this article centres them as valid forms of knowledge production, consistent with decolonial and Black feminist methodologies. This approach also resonates with McKittrick (2021) call to embrace creative and non-traditional forms of knowledge as legitimate, especially for those whose voices are often excluded from dominant academic frameworks. The following section highlights a selection of these visual narratives alongside analytic commentary that foregrounds their significance.

#### Image 2: *Blue Genes* (Grace, UK-RJ)


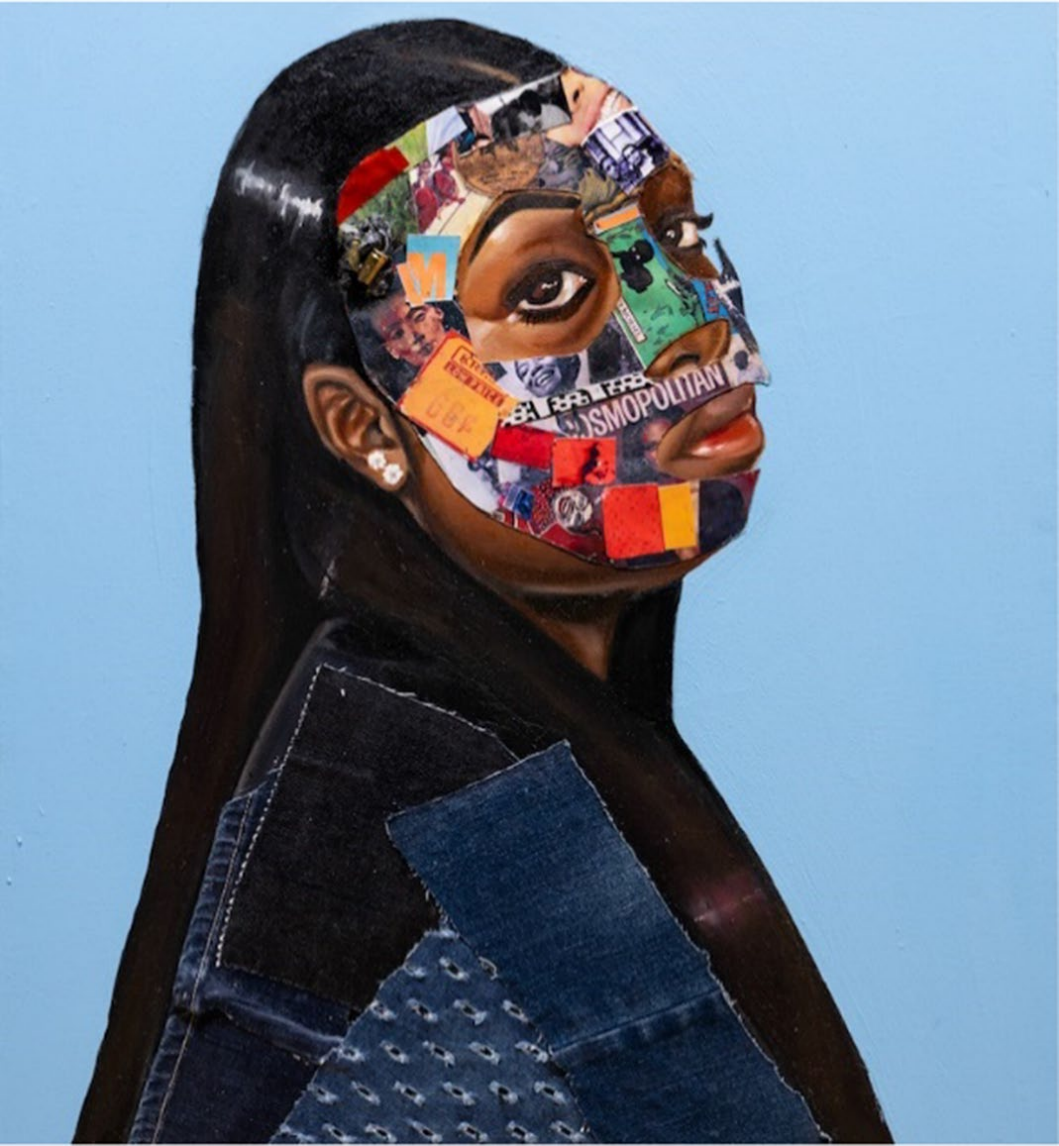
 Grace’s (UK-RJ) artwork *Blue Genes* plays on the dual meaning of “genes” and “blues,” invoking both intergenerational trauma and inherited racialised melancholy. She described the bright, multi-coloured patches in the image as representing “the ugliness of confinement disguised as something beautiful,” a metaphor that critiques the institutional tendency to perform allyship through aesthetic diversity while sustaining systems of exclusion (Ahmed, 2012). The artwork visually embodies the psychological toll of navigating white supremacist structures, aligning with Okello (2020) analysis of emotional preservation tactics employed by racially minoritised academics to survive hyper-surveillance and institutional scrutiny.

The collage elements reflect the fragmented yet resilient identities of Black women navigating academic spaces where their presence is often both hyper-visible and devalued. This resonates with Puwar (2004) notion of the “space invader,” where Black women are rendered as outsiders within predominantly white institutions. By selecting and interpreting this piece, Grace transforms *Blue Genes* into a critical lens through which systemic fatigue, racialised surveillance, and institutional gaslighting are exposed (Alexander-Floyd, 2015).

Drawing on Leavy (2018) work on visual methodologies, the use of collage here enables the articulation of complex, often unspoken realities—making the artwork not only a form of self-expression but also a powerful act of intellectual and emotional resistance. In this way, *Blue Genes* aligns with decolonial methodologies (Mignolo and Walsh, 2018) that validate lived experience, prioritise creative epistemologies, and resist dominant academic conventions that seek to silence marginalised voices.

#### The Black Dot and the Forever Tilting Seesaw (Lily, UK-RJ)


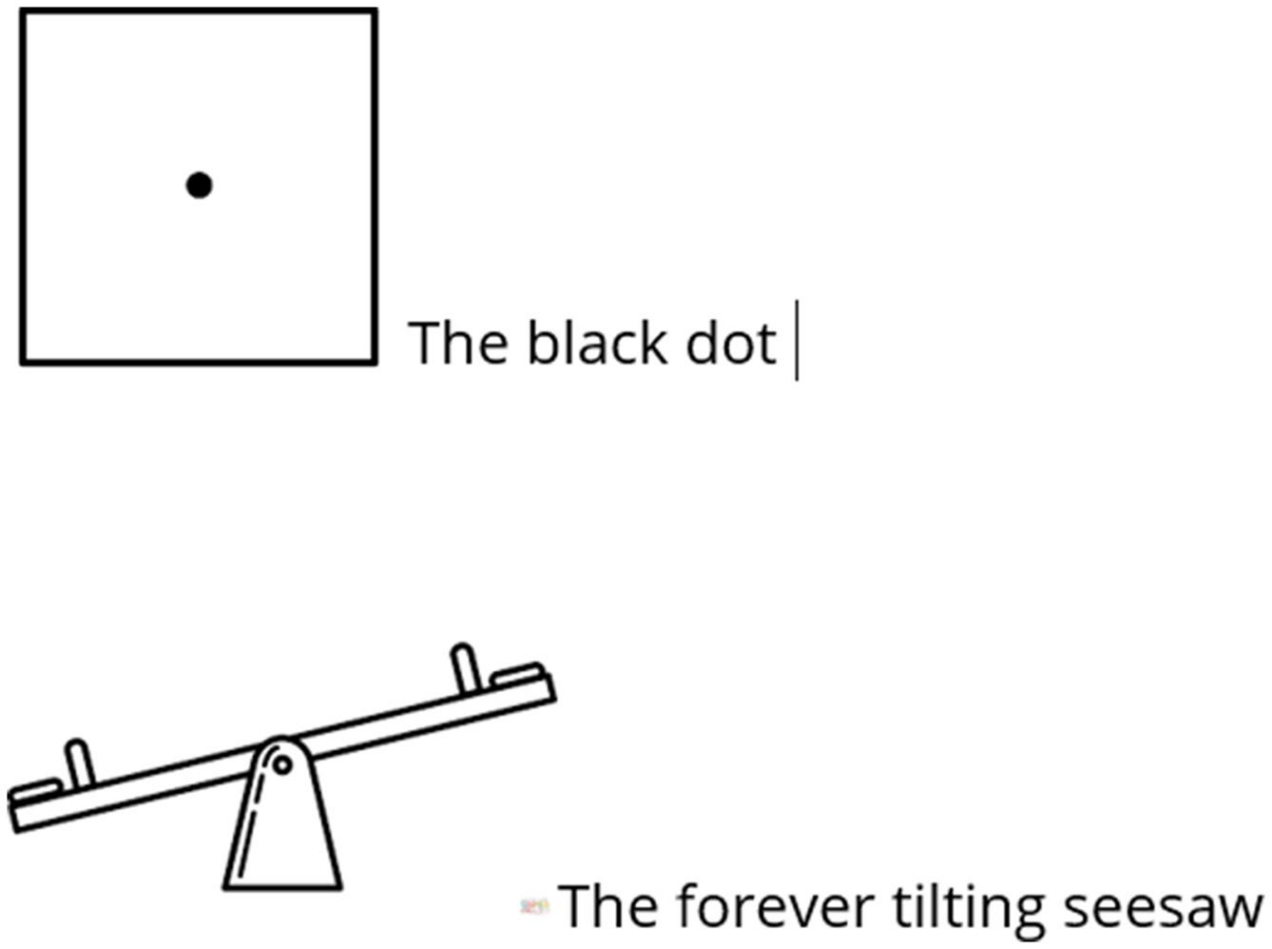
 Lily’s (UK-RJ) visual submissions, the Black Dot and the Forever Tilting Seesaw, powerfully convey the psychological strain and structural instability that Black and mixed-heritage women navigate in higher education. The Black Dot, with its stark contrast—a single black dot centred in a white square—offers a searing visual metaphor for institutional tokenism. Lily reflected that “the focus becomes the black dot and not the white space,” critiquing how institutions fixate on visible diversity while leaving the broader structures of whiteness unchallenged. This imagery highlights the hypervisibility of racialised identity: a singular presence spotlighted amidst a sea of presumed neutrality, echoing Joseph-Salisbury (2019) discussion of racialised embodiment in white institutional space.

The Forever Tilting Seesaw, by contrast, evokes the emotional volatility and precarity of navigating these environments. The image suggests an ongoing negotiation, between being “too light to be part of the Black community,” as Lily expressed, and yet never fully accepted into white-dominated spaces. This speaks directly to the tensions explored by Garrett (2025), who describes how mixed-heritage women in academia experience simultaneous racialisation and disqualification, often questioned for their authenticity. Together, the images enact a powerful duality: one focused on being singularised and exposed, the other on imbalance and instability.

Lily’s metaphors reflect the embodied consequences of institutional whiteness and the racialised burden of belonging. These visual artefacts act as counter-narratives that expose the affective labour of inhabiting spaces that were not built for Black or mixed-heritage women and challenge the superficiality of diversity discourses.

#### The mask (Celeste-RJ)


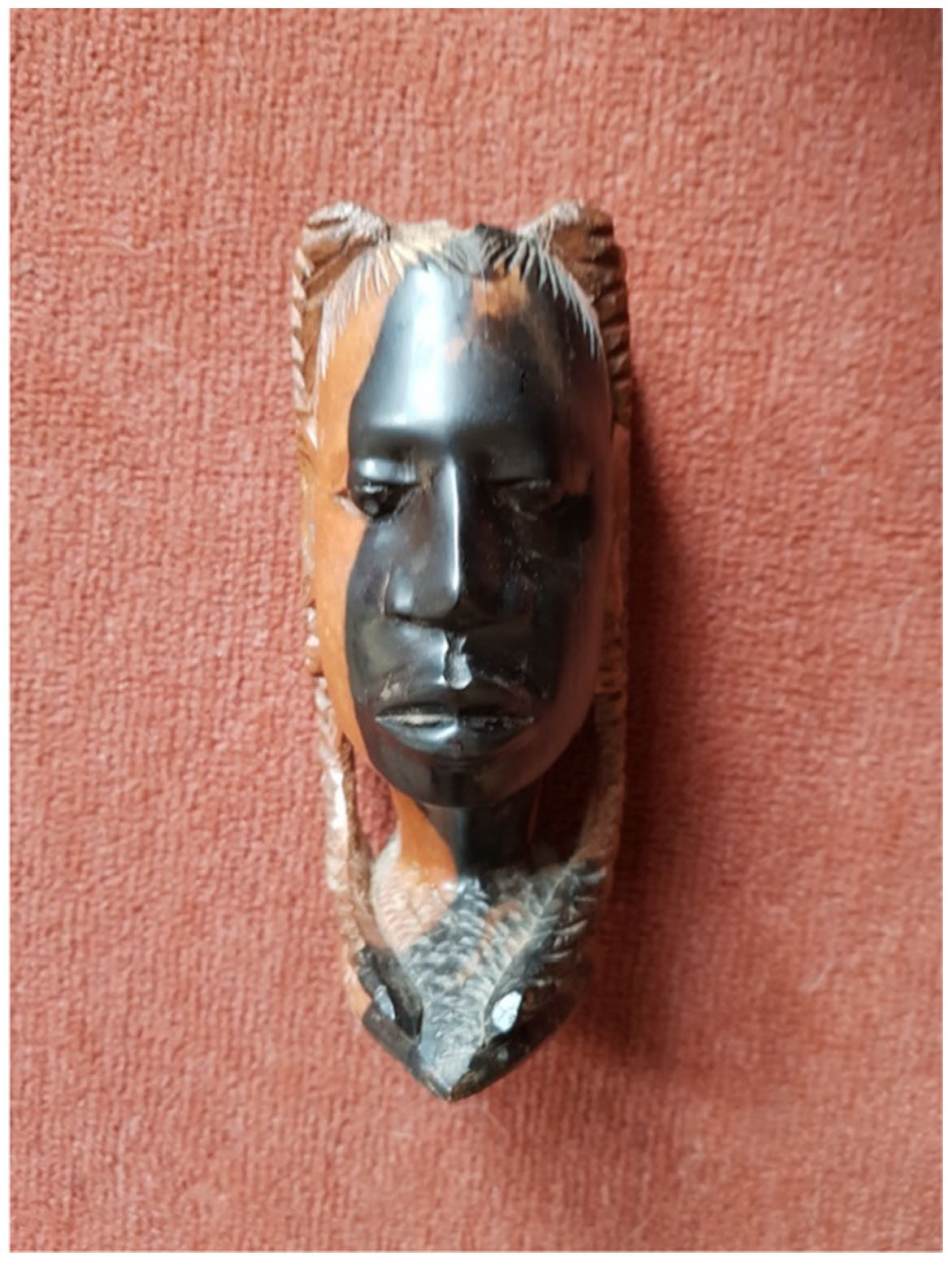
Celeste’s (UK-RJ) reflective journal includes a striking visual metaphor: a mask split in two, symbolising the duality of hypervisibility and erasure. While earlier sections of this article explore the emotional and institutional meanings behind this image, here it is important to foreground how the act of visual creation itself becomes a form of resistance. The left-facing side of the mask is textured and layered, representing assertion and visibility; the right side, by contrast, is subdued, marking the psychological toll of institutional silencing.

This image functions not merely as illustration but as epistemological intervention. In rendering institutional whiteness visually, Celeste offers a critique that is affective, embodied, and precise moving beyond the limits of textual description. The mask, then, is not just a personal symbol, but a visual theory of racialised conformity. This work builds on McKittrick (2021) call for unorthodox practices of belonging and expands Collins (2000) concept of the “outsider within.” While Collins originally used the term to describe the unique standpoint of Black women working within structures that marginalise them, I extend this idea by exploring how this positionality is expressed through visual and creative forms.

### Discussion of findings

This research demonstrates that storytelling and creative methodologies are not only tools of expression but strategies of decolonial praxis. In foregrounding Black women’s narratives, the study surfaces systemic patterns of exclusion while also revealing acts of resistance that challenge dominant epistemologies and institutional norms. These methodologies disrupt entrenched power structures while fostering spaces of equity, inclusion, and liberation. The recommendations outlined here expand upon the study’s findings and theoretical framework, particularly the use of Critical Race Theory, Black Feminist Thought, and decolonial methodologies as tools for reimagining university spaces.

### Theoretical framing and synthesis

Across UK and Canadian contexts, contributors’ narratives revealed a landscape defined by symbolic inclusion and emotional regulation, exposing institutional contradictions that demand performance without protection. These dynamics are well captured by Du Bois (1903) concept of double consciousness, which highlights the psychological toll of living in a world that renders Blackness hyper-visible in moments of surveillance or stereotype, while simultaneously erasing it from narratives of belonging. For the women in this study, this duality was not abstract, it lived in the expectation to smile through marginalisation, to mentor others while being passed over for leadership, and to represent diversity without institutional support. Their efforts to find ‘pockets of safety’ and create sister-networks were not just acts of survival but strategies of epistemic safety and resistance.

Intersectionality (Crenshaw, 1991) sharpens this dissonance. Neurodivergence, migrant identity, career stage, and colourism shaped how contributors were perceived, excluded, or occasionally celebrated. Microaggressions were far from minor; when situated within a context of structural underrepresentation, they formed a continuum of epistemic and psychological harm, reinforcing my framing of these acts as racial violence rather than microaggressions.

Black Feminist Thought further enriches this reading by foregrounding the “outsider within” (Collins, 2000), a role many contributors inhabited with painful familiarity. They were in the room, but not at the table. Their presence was requested for optics, yet their insights were disregarded in decision-making. This partial inclusion reinforced their marginalisation by forcing them to exist as symbols without power. In response, they sought out “pockets of safety” and cultivated relational forms of care, acts of resistance that became epistemic anchors amid the erosion of dignity and institutional hostility.

Across these experiences, contributors named a sense of institutional betrayal, a recurring theme when universities proclaimed their commitment to equity but failed to protect or promote those most impacted by inequality. This resonates with Ahmed (2012) critique of diversity work as often non-performative: used to signal inclusion while maintaining whiteness as the default. Contributors recognised that their presence was leveraged for branding, but not for transformation.

Yet amidst this harm, the contributors did not frame themselves as victims. Their strategies, naming joy, refusing to conform, seeking solidarity, speaking truth to power, represent an active reimagination of academic life. The theoretical frameworks at the core of this study, decolonial theory, Black feminist thought and CRT, help to situate these actions not as isolated acts of defiance, but as part of a global, diasporic struggle to create spaces of belonging, knowledge, and joy within institutions that were never built for them.

### Storytelling as decolonial praxis

Storytelling has proven to be a powerful mechanism for reclaiming agency and challenging systemic marginalisation. To elevate its role within higher education, institutions must integrate storytelling into academic curricula and research frameworks. The inclusion of storytelling as a pedagogical tool fosters spaces where lived experiences are not just validated but positioned as integral to the production of knowledge. For instance, contributors like Beauty in Flight (UK-RJ) shared how storytelling and poetry allowed her to articulate complex realities, transforming narratives of marginalisation into expressions of resistance and self-affirmation. Establishing digital and physical archives that preserve these narratives, with explicit consent to do so, would enable institutions to embed racially minoritised voices into their histories, ensuring that these accounts actively inform institutional policies and strategies.

### Creative methodologies and institutional transformation

To achieve meaningful transformation, universities must shift from performative commitments to actionable decolonial frameworks. This requires embedding anti-racist values into every level of governance, from hiring practices to curricular design. Representation alone is insufficient without systemic reforms that dismantle the power structures perpetuating exclusion. Creative methodologies, such as poetry, visual art, and performance, must also be embedded into institutional practices. Through this paper I demonstrated how such approaches offered contributors alternative means of expression that resisted the epistemic violence of traditional academic frameworks. Institutions should actively support creative research through funding opportunities, exhibitions, and workshops. Recognising creative outputs as legitimate scholarly contributions would disrupt the dominance of Eurocentric metrics that often marginalise Black women’s intellectual contributions. By reframing the metrics of academic success, universities can create environments where creative and community-based methodologies are equally valued alongside traditional paradigms of knowledge production.

### Networks of solidarity and transnational feminism

This study revealed how networks of solidarity, sisterhood, and mutual support act as essential sources of resilience and empowerment. Contributors like Yasmin and Natalie (both UK-C) highlighted the transformative impact of external and internal networks, describing how these spaces allowed them to navigate and resist systemic oppression within predominantly white institutions (Emejulu, 2022). Universities must prioritise creating formalised storytelling spaces, free from institutional scrutiny, where racially minoritised individuals can engage in narrative and creative practices that centre their well-being. Expanding cross-diaspora networks to connect Black women across regions would facilitate shared strategies for resistance while enhancing global solidarity.

## Conclusion and recommendations

This study has illuminated the complex, painful, and empowering realities of Black women working in higher education across the UK and Canada. It foregrounds the systemic nature of exclusion, manifesting through hypervisibility, emotional labour, symbolic diversity initiatives, and the silencing of resistance. These are not isolated experiences, but structurally embedded conditions sustained by institutional practices that centre whiteness as default. While universities often proclaim commitments to equity, such gestures are frequently non-performative (Ahmed, 2012), serving to manage optics rather than enact meaningful change.

Yet within these conditions, Black women continue to practise forms of resistance grounded in care, creativity, and community. The findings and theoretical frameworks in this study make clear that such creative, relational, and justice-oriented practices already exist within Black women’s academic lives. The recommendations that follow offer ways institutions can honour and sustain these practices, not through appropriation, but through redistributing power, embedding care, and transforming structural conditions to support epistemic and emotional justice.

To that end, I offer the following interconnected recommendations for transforming higher education from symbolic inclusion to structural equity. These actions are grounded in the practices already enacted by Black women in this study, practices that must be resourced, valued, and embedded at the institutional level:

Support collective voice and structural representation. Shift from individualised inclusion efforts toward sustainable, collective platforms that enable Black women to shape institutional policy and culture. Fund affinity groups, implement participatory review panels, and formalise consultation roles. Equity cannot exist without shared decision-making power.

Recognise and redistribute invisible labour. Acknowledge emotional care, mentoring, and EDI work within workload models and career progression frameworks. Co-develop trauma-aware wellbeing policies and create protected spaces for restoration—not as extras, but as essential infrastructure.

Embed joy and cultural grounding into institutional life. Treat Black joy not as symbolic celebration bust as a condition of safety and thriving. Joy should inform wellbeing strategies, be resourced through creative spaces, and be supported in ways that do not overburden those already marginalised.

Prioritise mentorship as epistemic and emotional care. Design reciprocal, relational mentorship programmes that foster intergenerational knowledge-sharing and psychological safety. Avoid overloading Black women with unpaid mentoring expectations.

Elevate storytelling and creative practice as scholarship. Fund and institutionalise storytelling, visual work, and performance as rigorous academic contributions. Establish accessible publishing and training pathways that affirm lived experience as legitimate knowledge.

Expand what counts as academic success. Revise evaluative metrics to include creative and community-based outputs central to decolonial and equity-driven research. Recognise non-traditional forms of excellence across disciplines and roles.

Higher education must become a space not only of access, but of transformation. The contributors to this study are already modelling alternative futures, through care, creativity, and joy that resist erasure. Institutions must meet this vision with structural courage: redistributing power, embracing relational accountability, and making room for ways of knowing long pushed to the margins.

To centre Black joy and storytelling is to move beyond recognising harm toward building spaces where Black women can live, lead, and thrive, on their own terms. The work of justice is not only to recognise harm, but to make joy possible.

## Data Availability

The raw data supporting the conclusions of this article will be made available by the authors, without undue reservation.
